# Shifts in the bacterial community composition along deep soil profiles in monospecific and mixed stands of *Eucalyptus grandis* and *Acacia mangium*

**DOI:** 10.1371/journal.pone.0180371

**Published:** 2017-07-07

**Authors:** Arthur Prudêncio de Araujo Pereira, Pedro Avelino Maia de Andrade, Daniel Bini, Ademir Durrer, Agnès Robin, Jean Pierre Bouillet, Fernando Dini Andreote, Elke Jurandy Bran Nogueira Cardoso

**Affiliations:** 1 Departament of Soil Science, “Luiz de Queiroz” College of Agriculture, ESALQ/USP, University of São Paulo, Piracicaba, São Paulo, Brazil; 2 University Centro-Oeste, UNICENTRO, Guarapuava, Paraná, Brazil; 3 Agricultural Research for Development, CIRAD, Montpellier, France; Nederlands Instituut voor Ecologie, NETHERLANDS

## Abstract

Our knowledge of the rhizosphere bacterial communities in deep soils and the role of *Eucalyptus* and *Acacia* on the structure of these communities remains very limited. In this study, we targeted the bacterial community along a depth profile (0 to 800 cm) and compared community structure in monospecific or mixed plantations of *Acacia mangium* and *Eucalyptus grandis*. We applied quantitative PCR (qPCR) and sequence the V6 region of the 16S rRNA gene to characterize composition of bacterial communities. We identified a decrease in bacterial abundance with soil depth, and differences in community patterns between monospecific and mixed cultivations. Sequence analysis indicated a prevalent effect of soil depth on bacterial communities in the mixed plant cultivation system, and a remarkable differentiation of bacterial communities in areas solely cultivated with *Eucalyptus*. The groups most influenced by soil depth were *Proteobacteria* and *Acidobacteria* (more frequent in samples between 0 and 300 cm). The predominant bacterial groups differentially displayed in the monospecific stands of *Eucalyptus* were *Firmicutes* and *Proteobacteria*. Our results suggest that the addition of an N_2_-fixing tree in a monospecific cultivation system modulates bacterial community composition even at a great depth. We conclude that co-cultivation systems may represent a key strategy to improve soil resources and to establish more sustainable cultivation of *Eucalyptus* in Brazil.

## Introduction

*Eucalyptus* is one of the most common genera in commercial forest plantations worldwide [1]. In Brazil there are 5.10 million hectares of planted *Eucalyptus*, and accordingly the species plays an important role in the country’s economy [2]. Most of the *Eucalyptus* plantations in Brazil are monocultures [2], which may cause several problems in soils such as an imbalanced nutrient content [3, 4]. A possible solution is the co-cultivation of *Eucalyptus* and leguminous trees in mixed systems [5–7] to provide an additional supply of nitrogen (N) for *Eucalyptus* [6, 8–10]. This strategy for N_2_ fixation is well described and is promoted by nodules found in *Acacia mangium* [11], the most commonly used legume in experimental stands of mixed plantations with *E*. *grandis* in Brazil [6, 12]. Co-cultivation also benefits plants by creating more heterogeneous systems, providing more bio-diverse sources of microbes, and supporting efficient selection in the rhizosphere [13].

Recent studies have indicated that there are benefits to the co-cultivation of *E*. *urophylla* or *E*. *grandis* with *A*. *mangium*, and revealed that this cultivation system affects the composition of the soil microbial community [4]. Furthermore, plant monocultures select more homogeneous bacterial communities in soil [14, 15]. Plants in forest plantations are able to select specific groups of microorganisms in the soil [16], but how this process takes place in communities along deep layers of tropical soils remains unknown, especially in areas with crop systems combining *Eucalyptus* and *Acacia*.

Some studies suggest that despite the importance of microorganisms to the soil microbiome, the influence of plants would be usually limited to the 0–20 cm soil layer [17–21]. However, since approximately 50% of the organic carbon in forest soils is stored below the uppermost 20 cm layers [22–24], it is of fundamental importance to investigate the effect of plants on soil microbiomes at deeper layers. Although microbial biomass and nutrient content are lower in layers deeper than 20 cm, the volume of the deep soil profile is very high particularly in the tropics. Thus, a large portion of the microbial community remains unexplored. These microbes may be of remarkable importance to forest plantations, where roots are deeper and reside in soils for a long time [25].

Here we sequenced the V6 hypervariable region of the 16S rRNA gene and performed quantitative PCR to characterize changes in the bacterial community along a deep profile of ferralsols cultivated with either monospecific or mixed stands of *E*. *grandis* and *A*. *mangium*. Our goal was to evaluate the bacterial soil community at an increasing depth and to examine its composition in monocultures and co-cultivated systems of *E*. *grandis* and *A*. *mangium*. Our hypotheses were as follows: i. The 16S rRNA gene should vary as a function of identity and abundance of the taxonomic groups, and as a function of depth. ii. The insertion of leguminous trees in co-cultivation systems with *Eucalyptus* should imply more heterogeneous bacterial community structures in the soil in comparison to monocultures.

## Materials and methods

### 2.1 Area cultivated with *Eucalyptus* and *Acacia*

The study was conducted at the Experimental Station for Forest Sciences of Itatinga, São Paulo State, Brazil (23°02’S 48°38’W, 860 m altitude). The soil is a ferralsol (FAO classification) [6] with sandy characteristics (~ 85% sand), low cation-exchange capacity and low nutrient content [6]. Long-term average rainfall is 1,395 mm (1990–2013), with a cold and relatively dry season from June to September, and a rainy and warm summer from September to March. Local topography is flat interspersed with gently rolling hills, which is typical of the western São Paulo plateau [6].

### 2.2 Sampling and soil characterization

Soil was sampled from the monospecific cultivation of *A*. *mangium* (treatment 100A) or *E*. *grandis* (treatment 100E) and from mixed stands of *A*. *mangium* and *E*. *grandis* (50A-50E). The mixed stand (50A-50E) was subdivided so that one half of the soil samples were taken from around *A*. *mangium* trees (treatment A(A+E)) and other half around *E*. *grandis* trees (treatment E(A+E)). Mixed stands had a 1:1 ratio of *A*. *mangium* and *E*. *grandis*. Spacing between trees was set at 3 x 3 m and plot areas at 30 x 30 m. Within each plot, we focused on areas of 18 x 18 m, in order to avoid bordering effects. We opened trenches and discarded edges in each of those plots [6]. Trenches measured 0.6 x 1.65 m and were 8m deep. We discarded approximately 25 cm of the external soil border from each trench to avoid cross-contamination between soil layers. Samples were collected in October 2013, when the trees were 4 years old and approximately 12 m high.

Five soil layers (0–100, 100–300, 300–500, 500–700, and 700–800 cm) were sampled from three trenches, one from each plot. From each soil layer, we collected and homogenized four subsamples to create composite samples [26]. The resulting 60 samples thus represented four treatments [100A, 100E, 50A-50E: A(A+E) and E(A+E)], three replicates, and five soil depths. From each sample, a 400-g soil portion (after sieving and air drying) was stored in plastic bags, kept at 4°C, and subsequently subjected to soil chemical and physical analyses, following the methodology proposed by Raij et al. [27] and by the Brazilian Agricultural Research Corporation [28]. Samples were placed in centrifuge tubes in the field and then kept at -80°C for DNA extraction and molecular analysis at the “Luiz de Queiroz” College of Agriculture.

### 2.3 DNA extraction

We extracted genomic DNA from 400 mg of soil using the PowerSoil*®* DNA Isolation kit (MoBio Laboratories, Carlsbad, CA, USA). Integrity of samples was assessed through 1.5% agarose gel electrophoresis with 1x TAE buffer (400 mM Tris, 20 mM acetic acid and 1 mM EDTA), subsequently stained with GelRed™ (0.5 μg mL^-1^), visualized and photo-documented under ultraviolet light (DNR–Bio Imaging Systems/MiniBis Pro).

### 2.4 Quantitative real-time PCR (qPCR)

The abundance of copies of the 16S rRNA gene (g soil^-1^) was determined using quantitative PCR (qPCR). The qPCR mixture (25 μL) contained 0.3 μL of each primer (0.4 mM) 341F (5’- CCTACGGGAGGCAGCAG -3’) and 534R (5’- ATTACCGCGGCTGCTTGG -3’), 12.5 μL of the fluorescent marker SYBR® Green PCR Master Mix 2x (Applied Biosystems®) and 1 μL of DNA template (approx. 50 μg mL^-1^), generating fragments of 193 base pairs [29]. Each reaction was duplicated, and we also included positive and negative control samples (free of DNA) to monitor for potential contamination. Samples were loaded into the StepOne™ Real-Time PCR System (Applied Biosystems^®^), and reactions were subjected to denaturation at 95°C for 3 min; 35 cycles of 94°C for 30 s, 55°C for 30 s, and 72°C for 30 s; and a final extension of 72°C for 45 s [30]. Amplification specificity was confirmed by melting curve analyses obtained from serial dilutions (10^−2^ to 10^−8^ gene copies μL^-1^). Consistency in Ct values and consequently in quantification values validated our approach. Amplification reaction efficiency was 0.98, with R^2^ values higher than 0.98 for all calibration curves.

### 2.5. Sequencing of the bacterial gene 16S rRNA and data analysis

To analyse the structure and composition of the bacterial community, the V6 region of the 16S rRNA gene was amplified with primers A-967F and B-1046R [31]. Primers were amended with adapters for the Ion Torrent Personal Genome Machine System sequencing (PGM) (Life Technologies, USA). Primer A-967F received an additional 5-bp tag to identify sequences from each sample. PCR reactions were performed following an initial denaturation step at 94°C for 3 min; 30 cycles at 94°C for 30 s, 57°C for 45 s, and 72°C for 1 min; and a final extension of 72°C for 2 min [31]. PCR products were purified with the ChargeSwitch® PCR Clean-Up kit (Life Technologies) and subsequently sequenced by the Ion Torrent Personal Genome Machine System (PGM) available at the Microbiology Laboratory of the National Centre for Environmental Research, Assessment and Impact Evaluation—EMBRAPA (Jaguariúna, São Paulo, Brazil).

Sequence analysis was conducted using QIIME (Quantitative Insights into Microbial Ecology) software [32]. Sequences were separated by sample according to their 5-bp tags. Adapter and primer sequences were removed, and low-quality sequences (Q<20) were discarded [33]. Obtained data were rarefied at 14,404 sequences per sample to avoid bias caused by the different numbers of individuals sampled from each soil core. The resulting sequences were grouped into operational taxonomic units (OTUs) based on 97% similarity using the UCLUST method [34]. Representative sequences of each OTU were subjected to taxonomic analysis through the PYNAST method against the Greengenes database [32]. Sequence datasets were uploaded to the MG-RAST (Metagenomics Analysis Server) databank (accession number 265089).

### 2.6 Statistical analyses

Data were examined for homogeneity of variances and normal distribution. Furthermore, the dataset was subjected to ANOVA and mean comparisons by Tukey’s test (p<0.05). Bacterial community similarity was represented by Principal Coordinate Analysis (PCoA), using the Bray-Curtis dissimilarity metric distance after Hellinger’s standardization implemented with the software Primer-E 6.0 [35, 36]. To test whether bacterial composition differed between pure *Eucalyptus* and co-cultivation with *Acacia*, we applied the PERMANOVA statistical test. [37, 38]. The bacterial phyla and classes most contributing to differences among treatments were determined by the SIMPER test (Similarity percentages species contributions) using the Chord algorithm from the PAST software [38].

## Results

### 3.1 Soil properties

In general, soil pH values slightly increased with depth but remained at a value of 5.5 in most of the assessed cultivations and layers. When comparing the first soil layer (0–100 cm) with all the other layers, total N was significantly higher in treatments E(A+E) (1649.7 mg kg^-1^) and A(A E) (1641.9 mg kg^-1^). Treatment 100E presented the lowest total N content (1334.7 mg kg^-1^) (S1 Table). The mean values of available P (in mg kg^-1^), K^+^, Ca^2+^, Mg^2+^, and Al^3+^ (in mmol_c_ kg^-1^) in the 0–100 cm layer were respectively 2.3, 0.4, 2.0, 2.1, and 7.5, showing no significant difference among the treatments (S1 Table). Most of the abovementioned nutrients were below the detection limit in the layers below 100 cm, making it impossible to determine the exact content in the sample. Even in surface layers (0–100 cm) the soil showed a low percentage of the sum of bases and consequently limited values of base saturation (%V) below 50%. Organic matter (OM) values decreased with depth from 12.25 to less than 4.0 g kg^-1^ (average values of the treatments) from layers 0–100 to 700–800 cm. However, treatment 100E showed the highest OM values in all analysed soil layers (S1 Table). In particle size analysis, soil samples from all layers were classified as medium-sandy except for samples from the *A*. *mangium* cultivation classified as sandy between 100 and 300 cm (S1 Table).

### 3.2 Abundance of the 16S rRNA gene along soil depth and among treatments

**Fig 1:**
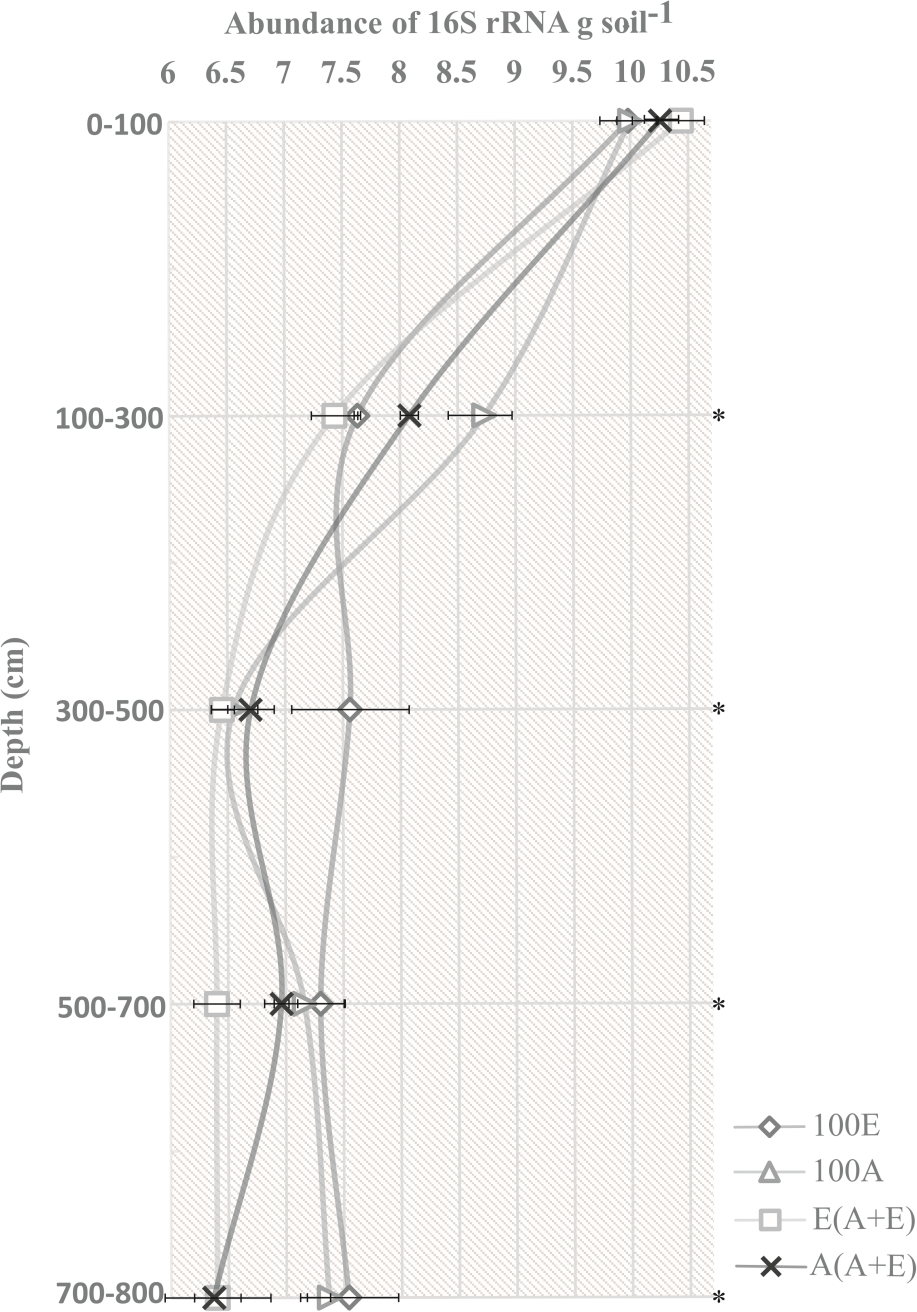
Abundance (n = 3) of the 16S rRNA gene. Treatments: 100A (*A*. *mangium* in a monospecific plantation system); A(A+E) (mixed plantation of *A*. *mangium* and *E*. *grandis*, with sampling at the *Acacia* base); 100E (*E*. *grandis* in a monospecific plantation system); and E(A+E) (plantation of *A*. *mangium* and *E*. *grandis*, with sampling at the *Eucalyptus* base). Asterisks indicate significant differences (p<0.05) between treatments.

There was a significant reduction in the abundance of the bacterial 16S rRNA gene with increasing soil depth, with log copies (g soil^-1^) ranging from 10.1 to 10.4 at 0–100 cm and 7.37 to 6.38 at 700–800 cm (p<0.05). We found no significant changes in the abundance of the 16S rRNA gene across treatments in samples from the surface layers (0–100 cm). Nevertheless, we found higher amounts of the target gene from 0 to 300 cm for treatments including *Acacia*, either in monospecific cultivation (100A, log copies g soil^-1^ = 8.73) or in mixed cultivation (A(A+E), log copies g soil^-1^ = 8.08) (p<0.05). Considering the layers of soil below 300 cm and down to 800 cm, the monocultures (100E and 100A) had a higher abundance of the 16S rRNA gene than other treatments (p<0.05). However, treatment 100E stood out from the others, showing the highest mean of log copies of the 16S rRNA genes (g soil^-1^): 100E = 7.58, whereas 100A = 7.02, E(A+E) = 6.20, and A(A+E) = 6.31 (Fig 1).

### 3.3 Bacterial community structures and composition

**Fig 2:**
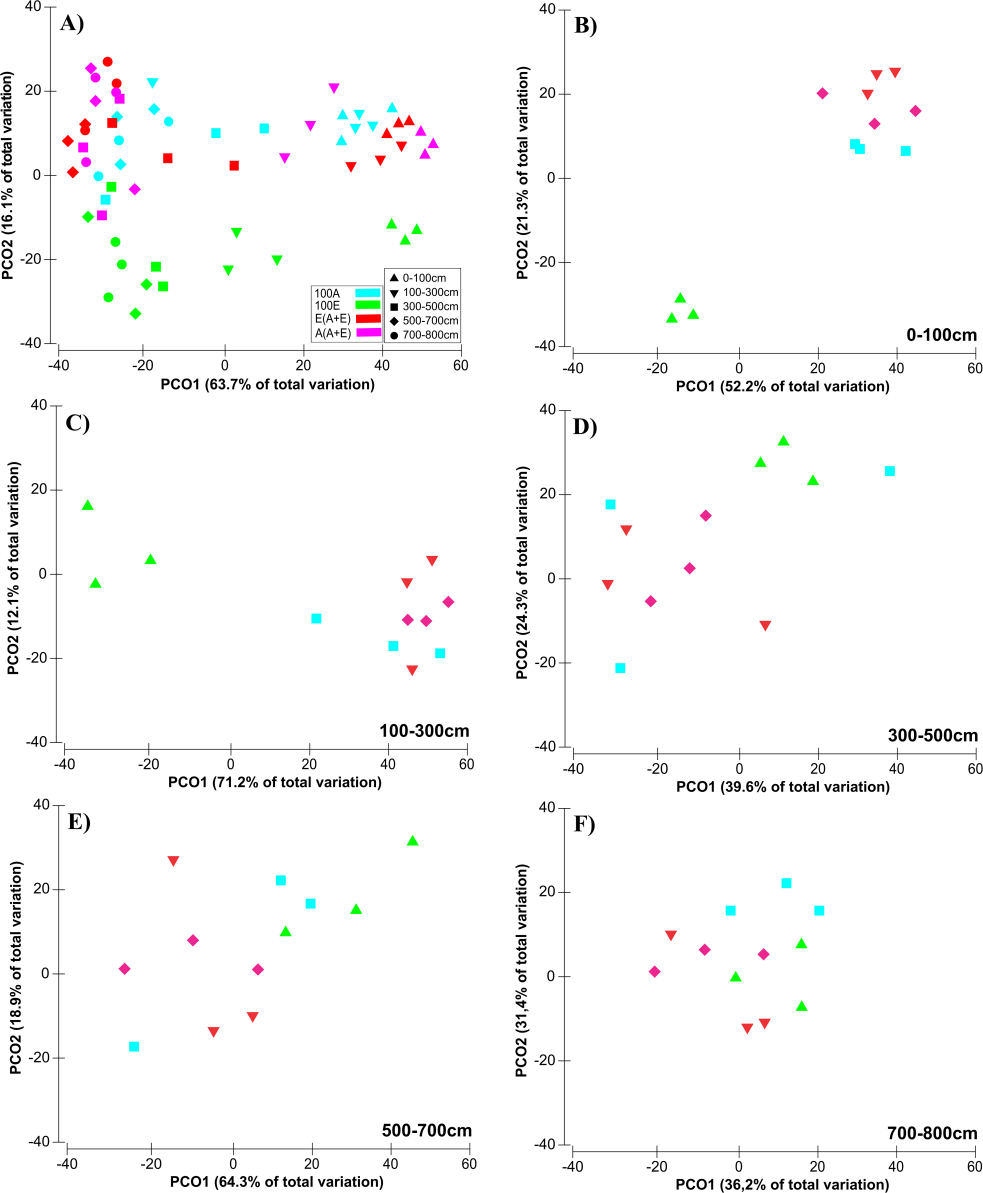
Principal coordinate analysis (PCoA) based on an OTUs matrix obtained in sequencing 16S (rRNA) through the Ion torrent platform. Groups based on the Bray-Curtis algorithm. 100A: *A*. *mangium* in a monospecific plantation system; A(A+E): mixed plantation of *A*. *mangium* and *E*. *grandis* with sampling at the *Acacia* base; 100E: *E*. *grandis* in a monospecific system; and E(A+E): mixed plantation of *A*. *mangium* and *E*. *grandis* with sampling at the *Eucalyptus* tree base. (A) refers to a total PCoA including all soil layers (0 to 800 cm) and all treatments; (B) refers to a soil layer depth between 0 and 100 cm, whereas (C) evaluates between 100 and 300 cm, (D) between 300 and 500 cm, (E) between500 and 700 cm and (F) from700 to 800 cm.

Structure and composition of the general bacterial community also exhibited clear and significant differences across soil layers and treatments (Fig 2A, S1 Fig).

A principal coordinate analysis revealed a first separation (PCO 1 = 63.7%) corresponding to the influence of soil depth, separating shallow soil samples (0–100 cm) from the others (below 100 cm). Accordingly, the PERMANOVA test showed significant separation (R = 3.22; p = 0.0003) (Fig 2A). The major bacterial groups responding to differences in soil depth were the phyla *Acidobacteria* and *Verrucomicrobia* prevalent in the shallow layers of 0–100 cm (S1 Fig, S3 Table) (p<0.05). They were followed by an increase in the frequency of phyla *Proteobacteria*, *Firmicutes*, and *Bacteroidetes* at a greater depth. The relative abundance of *Proteobacteria*, for instance, was twice as high in the 500–700 cm and 700–800 cm layers as in the 0–100 cm layer. Phylum *Firmicutes* showed almost four times higher abundance in deep layers than in the surface layer. The *Acidobacteria* community was significantly less prevalent at greater depths, at 38% in the 0–100 cm layer and 2.9% in the deepest layer (700–800 cm).

The secondary separation (PCO 2 = 16.1%) shows a sample cluster originating from treatments 100A, A(A+E), and E(A+E) that is distinct from samples 100E based on the PERMANOVA results (R = 1.97; p = 0.0001) (Fig 2A). We found that the surface (0–100 and 100–300 cm) samples of treatment 100E had a clearly different bacterial community pattern compared with those from areas 100A, A(A+E), and E(A+E) (Fig 2A**–**2C). Moreover, from top to bottom communities became more dispersed and less separated from each other. Thus, similar patterns were seen between areas at depths below 300 cm (Fig 2D–2F). Furthermore, the A(A+E) and E(A+E) samples shared many bacterial groups with the *A*. *mangium* monoculture (Fig 2A–2D).

These major groups occurred in distinct ordinations across treatments. *Proteobacteria* was the most abundant phylum in samples from treatments A(A+E) and E(A+E) or in the monoculture of *Acacia mangium* (100A), while in the Eucalypt monoculture (100E) the prevalent phylum was *Firmicutes* (S1 Fig, S2 Table) (p<0.05). The SIMPER test discriminated the main groups that contributed to the shifts in bacterial communities between the 100E treatment and the 100A, A(A+E), and E(A+E) treatments (Table 1).

**Table 1:** Maximum contribution based on the SIMPER test of the bacterial phyla and classes. Results point to a significant separation of treatment 100E (Eucalypt monoculture) from the other cultivation systems.

Phyla	%	Classes	%
*Proteobacteria*	27.34	*Betaproteobacteria*	17.31
		*Alphaproteobacteria*	4.22
		*Gammaproteobacteria*	4.01
		*Deltaproteobacteria*	1.8
*Firmicutes*	18.73	*Bacilli*	15.93
		*Clostridia*	2.8
*Acidobacteria*	19.94	*Solibacteres*	2.87
		*Chloracidobacteria*	0.45
		Others	16.62
*Bacteroidetes*	11.16	*Flavobacteria*	7.21
		*Sphingobacteria*	3.09
		*Saprospirae*	0.24
		*Cytophagia*	0.18
		*Bacteroidia*	0.82
*Actinobacteria*	4.20	*Actinobacteria*	3.40
		*Acidomicrobia*	0.26
		*Rubrobacteria*	0.2
		*Thermoleophilia*	0.34
*Cyanobacteria*	2.1	*Oscillatoriophycideae*	1.93
		Others	0.17
*Verrucomicrobia*	2.32	*Spartobacteria*	1.70
		*Pedosphaerae*	0.62
*Chloroflexi*	1.79	*Ktedonobacteria*	1.31
		*Anerolineae*	0.28
Others	12.42		

In this context, phylum *Proteobacteria* (26.4%) contributed the most to the distinction between the above-mentioned treatments. Within the phylum *Proteobacteria*, *Betaproteobacteria* (16.6%) was the most representative class, expressing the highest percentage of dissimilarity among the tested patterns, followed by *Alphaproteobacteria* (4.3%), *Gammaproteobacteria* (3.6%), and *Deltaproteobacteria* (1.7%) (S2 Fig, Table 1, S4 and S5 Tables). A large fraction of the variation between the treatments can be explained by the presence of the phyla *Firmicutes* (19.6%), *Acidobacteria* (19.3%), and *Bacteroidetes* (12.6%) in the 100E treatment, which were represented by the classes *Bacilli* and *Flavobacteria*, while other phyla and classes, as well as unclassified sequences, totalled 11.3% of the group variations between the treatments.

## Discussion

The bacterial community inhabiting the surface and subsurface of forest soils plays crucial roles in biogeochemical cycles [39, 40]. Although the physical-chemical soil attributes did not present significant differences among treatments (with the exception of the total mineral N), bacterial community structure presented a clear differentiation in the abundance and composition of the rRNA gene along soil layers in response to the established treatments. We did not find any published analysis of the abundance of the 16S rRNA gene in soil layers below a 300-cm depth and thus our inferences regarding deeper soil layers (down to 800 cm) rely exclusively on our own insights.

Previous studies in other plant systems have shown clear changes in the microbial community abundance and composition in soil layers of medium depth. Some authors have stated that the low carbon quantity available in deeper horizons is the prevalent modulating factor for a reduced bacterial community in the subsurface layers [16, 26]. The decrease in the bacterial community seems to be related to deeper soil layers, which may act as ecological filters, reducing the overall biomass [13]. Thus, the subsurface environment might select for microbes that can survive under reduced energy sources, limiting the development of highly complex bacterial communities; alternatively, the subsurface soil life must have distinct substrate utilization, leading to the niche colonization of a more specialized and slow-growing bacterial community structure [17, 19, 23, 41]. The shift in the bacterial community along soil layers is not exclusively related to the quantity of bacterial gene copies.

We found a shift in the relative abundance of the phyla *Acidobacteria* and *Proteobacteria*. While *Acidobacteria* predominated in the surface layer of the treatments, subsurface layers (below 300 cm) were dominated by an exponential increase in the relative abundance of the phylum *Proteobacteria*, making it the most abundant phylum in the subsurface. In an investigation of the bacterial structure of rice crops, we had previously found increased numbers of the phylum *Proteobacteria* in soil layers down to 300 cm in depth [20]. This result suggests that the members of the phylum *Proteobacteria* can survive in a variety of soil types, including those that present higher pH values [42], those that are acidic and those that have a low availability of nutrients [43]. The greatest decrease in abundance of *Acidobacteria* with increased depth is probably due to the aerophilic and acidophilic characteristics of the microorganisms that compose this phylum. We found a small increase in soil pH values with increased soil depth (S1 Table), which may be a factor discouraging the presence of *Acidobacteria* [16, 44]. The abundance of *Firmicutes* also showed high peaks in deep soil layers, mainly between 300 and 500 cm, consistently with the findings reported by Li et al. [20] for the subsoil layer. This phylum includes the class *Bacilli*, which is resistant to pH variations in the soil [45]. The prevalence of the phyla *Proteobacteria*, *Acidobacteria*, *Actinobacteria*, and *Bacteroidetes* was repeatedly reported, and we previously attributed this dominance to the acidic nature of forest soils [40, 46, 47]. *Actinobacteria*, in turn, are K-strategists, slow growing and have a marked ability to grow in oligotrophic environments, in addition to being drought-tolerant [48]. These considerations, and the fact that the phylum *Acidobacteria* is still unexplored due to its difficulty to be cultivated in laboratory conditions, indicate the importance of more detailed studies of these groups and their community structures and roles in forest ecosystems [40, 49, 50].

With regard to bacterial community structure, we found great differences even between the surface soil samples (0–100 cm) of the *Eucalyptus* stand and the three other treatments (Fig 2A). This finding suggests a more potent “rhizospheric effect” caused by *Acacia*, probably related to its particular supply of carbon exuding from the roots. Another possible reason for this influence of *A*. *mangium* is its great acidification of the rhizosphere due to an intense absorption of cations and a constant liberation of H+ ions from the roots, which may contribute to the modification of the bacterial community structures in this environment [4]. Moreover, *Acacia* is a leguminous N_2_-fixing tree, and the resulting increased N availability may be the most important factor modulating the soil microbiome. Furthermore, N fixation is an important strategy for replenishing N in the soil and for the development of *Eucalyptus* [11] and considered the most important stimulus for improving soil C and N concentrations, stimulating the development of the nitrifying community, and optimizing intercrop growth [4, 12]. N fixation by *A*. *mangium* is due to an association between this plant and the N-fixing bacterium *Rhizobium* of the phylum *Proteobacteria* [4, 8]. However, our results indicate a much greater enrichment by *Betaproteobacteria* than by *Alphaproteobacteria*. Thus, we suggest that *A*. *mangium* may be another tropical leguminous tree modulated by *Betaproteobacteria*, alongside *Ensifer* or *Burkholderia* among others [51].

Generally, bacterial groups at the soil surface (0–100 cm) were the most compacted, possibly as a result of greater daily and seasonal variation. However, in soil layers below 300 cm we found a smaller allocation of OM, a limiting condition for many bacterial groups [52]. The lower variation of the chemicals attributes in deep soil suggests that there is little microbial genetic variability in deeper horizons [19]. Such conditions can cause genetic and metabolic processes to occur more randomly, leading to the formation of scattered and more dissimilar clusters. Thus, we suggest that the possible dominance effect of *A*. *mangium* on the bacterial community is limited to the surface layers of the soil.

Analysing results from the cultivation of *Eucalyptus* and leguminous tree intercrops, some authors have detected higher productivity in intercropped stands, a feature correlated with nitrogen-fixing ability, biomass production, nutrient cycling, and other benefits [53]. Corroborating these statements, we can add that intercropping plays a crucial role in shifts of the bacterial community structure and composition in comparison to monoculture. The diversification of the stand may help the rhizosphere to acquire new bacterial groups [4]. Changing environmental conditions are linked to changes in profiles of soil microbial communities in response to different management systems [4, 53, 54] and to changes imposed by different plant species [20]. The diversity of plant species [55–57] may cause a variability of C compounds to be released into rhizosphere ecosystems [58], rooting zone depletion [59], litter deposition, diffusion of allelopathic compounds [60, 61], and even effects on soil pH, humidity, and nutrient levels [62, 63]. Therefore, in light of our data we must assume that the co-cultivation of *E*. *grandis* with *A*. *mangium* is a successful methodology promoting the diversification and increase in functionality of the rhizosphere microbiome.

*Proteobacteria* may play a key role in structuring the bacterial community in soil, mainly due to the greater abundance of this phylum in the treatments containing leguminous trees (S2 and S4 Tables). N is one of the most important mineral nutrients, establishing healthy plant growth and rhizosphere bacteria modulation. This nutrient seems to be highly influenced by the dynamics resulting from insertion of a new plant species, resulting in modified bacterial community structure in intercropped plantations [4]. Many authors have demonstrated enhanced N availability in intercropped systems [5, 7, 8, 12], which is key to achieving soil microbial management, acting as a powerful microbial tool and thereby contributing to a better understanding of how to obtain ecological intensification in forests.

Our data suggest that it is possible to “manage” the soil bacterial community by using the adopted tree cultivation system, thereby increasing inherent gains in plantations of interest. This is relevant since microbial groups of interest can be introduced into soils as a result of associations between forest trees. This can give rise to a system of ecological intensification where both economic gain (genus *Eucalyptus*) and ecological interest (genus *Acacia*) are found in the same agroecosystem, with improved productivity and sustainability [4]. Our study pioneers the evaluation of bacterial communities in soil layers down to a depth of 800 cm in mixed plantations of *Eucalyptus* and *Acacia*, presents initial insights into the rhizosphere bacterial community structure, and will be complemented by our ongoing research.

## Conclusions

We verified the influence of the deep-soil and forest-tree composition on soil bacterial communities. In the co-cultivation of *E*. *grandis* and *A*. *mangium*, the legume exerted a greater influence on the soil bacterial community. Moreover, the soil bacterial community tends to be modulated by soil depth, and as a result it is possible to verify the formation of specific groups in each studied soil layer as a mechanism of plant recruitment. However, in deeper soil horizons, we could not detect typical and distinct rhizosphere communities. The major responsive bacterial groups were *Proteobacteria* and *Acidobacteria* (more frequent in samples between 0–100 and 100–300 cm). The major bacterial groups differentially displayed in the monospecific cultivation of *Eucalyptus* were *Firmicutes* and *Proteobacteria*. The use of mixed forests of *Eucalyptus* with leguminous trees emerges as a promising alternative for sustainability, and should be recognized as a highly important strategy for the conservation of soil biodiversity.

## Supporting information

S1 Table Chemical and physical properties of soil at various depths and in the different plantation systems. (DOCX)

S2 Table Average abundance (n = 3) of bacterial phyla across treatments. 100A (*A*. *mangium* in a monospecific plantation system); A(A+E) (mixed plantation of *A*. *mangium* and *E*. *grandis*, with sampling at the *Acacia* base; 100E (*E*. *grandis* in a monospecific plantation system); and E(A+E) (plantation of *A*. *mangium* and *E*. *grandis*, with sampling at the *Eucalyptus* base). “Others” represents unclassified sequences.
(DOCX)

S3 Table Average abundance (n = 3) of bacterial phyla in the soil layer. 100A (*A*. *mangium* in a monospecific plantation system); A(A+E) (mixed plantation of *A*. *mangium* and *E*. *grandis*, with sampling at the *Acacia* base; 100E (*E*. *grandis* in a monospecific plantation system); and E(A+E) (plantation of *A*. *mangium* and *E*. *grandis*, with sampling at the *Eucalyptus* base). “Others” represents unclassified sequences.
(DOCX)

S4 Table Average abundance (n = 3) of bacterial classes across treatments. 100A (*A*. *mangium* in a monospecific plantation system); A(A+E) (mixed plantation of *A*. *mangium* and *E*. *grandis*, with sampling at the *Acacia* base; 100E (*E*. *grandis* in a monospecific plantation system); and E(A+E) (plantation of *A*. *mangium* and *E*. *grandis*, with sampling at the *Eucalyptus* base). “Others” represents unclassified sequences.
(DOCX)

S5 Table Average abundance (n = 3) of bacterial classes in the soil layer. 100A (*A*. *mangium* in a monospecific plantation system); A(A+E) (mixed plantation of *A*. *mangium* and *E*. *grandis*, with sampling at the *Acacia* base; 100E (*E*. *grandis* in a monospecific plantation system); and E(A+E) (plantation of *A*. *mangium* and *E*. *grandis*, with sampling at the *Eucalyptus* base). “Others” represents unclassified sequences.
(DOCX)

S1 Fig Relative abundance (n = 3) of bacterial phyla across treatments. 100A (*A*. *mangium* in a monospecific plantation system); A(A+E) (mixed plantation of *A*. *mangium* and *E*. *grandis*, with sampling at the *Acacia* base); 100E (*E*. *grandis* in a monospecific plantation system); and E(A+E) (plantation of *A*. *mangium* and *E*. *grandis*, with sampling at the *Eucalyptus* base). “Others” represents unclassified sequences. Asterisks indicate significant differences (p<0.05) between treatments.
(PNG)

S2 Fig Relative abundance (n = 3) of bacterial classes across treatments. 100A (*A*. *mangium* in a monospecific plantation system); A(A+E) (mixed plantation of *A*. *mangium* and *E*. *grandis*, with sampling at the *Acacia* base); 100E (*E*. *grandis* in a monospecific plantation system); and E(A+E) (plantation of *A*. *mangium* and *E*. *grandis*, with sampling at the *Eucalyptus* base). “Others” represents unclassified sequences.
(PNG)
