# Supplementary material for: Shifts in the bacterial community composition along deep soil profiles in monospecific and mixed stands of Eucalyptus grandis and Acacia mangium
Source: PLoS One. 2017 Jul 7;12(7):e0180371. doi: 10.1371/journal.pone.0180371 (PMC5501519; doi:10.1371/journal.pone.0180371)
Supplement: S1 Table — (DOCX) [file pone.0180371.s001.docx]

|  | Depth | pH | Total N | P** | K^+^ | Ca^2+^ | Mg^2+^ | Al^3+^ | H+Al^***^ | Sand | Silt | Clay^1^ | OM^2^ |
| --- | --- | --- | --- | --- | --- | --- | --- | --- | --- | --- | --- | --- | --- |
|  | (cm) | (KCl) | mg kg^-1^ | g.kg^-1^ | ----------------mmol_c_.kg^-1^---------------- | | | | | -------------g.kg^-1^------------- | | | |
| 100E | 0-100 | 3.9 | 1334,7 c | 2.1 | 0.4 | <2 | 2 | 6 | 32 | 834 | 16 | 150 | 16 |
|  | 100-300 | 4.1 | 9.2 ^NS^ | 1 | <*0.3 | <2 | <1 | 1 | 20 | 793 | 32 | 175 | 9 |
|  | 300-500 | 5.2 | 13.4  ^NS^ | 1 | <0.3 | <2 | <1 | <1 | <10 | 756 | 29 | 216 | 4 |
|  | 500-700 | 5.2 | 8.15  ^NS^ | 1 | <0.3 | <2 | <1 | <1 | 10 | 736 | 33 | 231 | <4 |
|  | 700-800 | 4.9 | <0,3  ^NS^ | 1 | <0.3 | <2 | <1 | <1 | 15 | 734 | 28 | 238 | <4 |
| 100A | 0-100 | 3.0 | 1476,2 b | 2.4 | <0.3 | <2 | 1 | 7 | 39 | 846 | 29 | 125 | 13 |
|  | 100-300 | 4.3 | 58.3 ^NS^ | 1 | <0.3 | <2 | <1 | 3 | 19 | 792 | 20 | 188 | 5 |
|  | 300-500 | 5.1 | 13,7  ^NS^ | 1 | <0.3 | <2 | <1 | <1 | 11 | 766 | 21 | 213 | <4 |
|  | 500-700 | 5.4 | 2,1  ^NS^ | 1 | <0.3 | <2 | <1 | <1 | <10 | 740 | 21 | 239 | <4 |
|  | 700-800 | 5.0 | <0,8  ^NS^ | 1 | <0.3 | <2 | <1 | <1 | <10 | 733 | 16 | 251 | <4 |
| E(A+E) | 0-100 | 3.7 | 1649.7 a | 2.3 | <0.3 | <2 | 2 | 8 | 36 | 893 | 29 | 76 | 10 |
|  | 100-300 | 4.0 | 89.7  ^NS^ | <1 | <0.3 | <2 | <1 | 3 | 22 | 790 | 30 | 170 | 5 |
|  | 300-500 | 5.0 | 42.3  ^NS^ | <1 | <0.3 | <2 | <1 | <1 | 11 | 778 | 20 | 205 | <4 |
|  | 500-700 | 5.2 | 7,8  ^NS^ | <1 | <0.3 | <2 | <1 | <1 | 10 | 730 | 14 | 217 | <4 |
|  | 700-800 | 4.9 | <1.0 ^NS^ | <1 | <0.3 | <2 | <1 | <1 | 10 | 751 | 22 | 224 | <4 |
| A(A+E) | 0-100 | 3.1 | 1641.9 a | 1.1 | <0.3 | <2 | 2 | 7 | 38 | 896 | 28 | 75 | 10 |
|  | 100-300 | 4.3 | 97.7  ^NS^ | <1 | <0.3 | 3 | <1 | 2 | 22 | 792 | 32 | 176 | 5 |
|  | 300-500 | 5.0 | 44.1  ^NS^ | <1 | <0.3 | <2 | <1 | <1 | 11 | 777 | 21 | 202 | <4 |
|  | 500-700 | 5.1 | 4.6  ^NS^ | <1 | <0.3 | <2 | <1 | <1 | <10 | 772 | 15 | 213 | <4 |
|  | 700-800 | 4.9 | <3,0  ^NS^ | <1 | <0.3 | <2 | <1 | <1 | <10 | 749 | 25 | 226 | <4 |

Table S1. Chemical and physical properties of soil at various depths and in the different plantation systems.

^1^ With dispersant – NaOH

^2^ OM – Organic matter.

<* Below detection limit

** Available P (P-Resin).

*** Potential acidity
