# Supplementary material for: Shifts in the bacterial community composition along deep soil profiles in monospecific and mixed stands of Eucalyptus grandis and Acacia mangium
Source: PLoS One. 2017 Jul 7;12(7):e0180371. doi: 10.1371/journal.pone.0180371 (PMC5501519; doi:10.1371/journal.pone.0180371)
Supplement: S2 Table — 100A (A. mangium in a monospecific plantation system); A(A+E) (mixed plantation of A. mangium and E. grandis, with sampling at the Acacia base; 100E (E. grandis in a monospecific plantation system); and E(A+E) (plantation of A. mangium and E. grandis, with sampling at the Eucalyptus base). “Others” represents unclassified sequences. (DOCX) [file pone.0180371.s002.docx]

Table S2. **Average** **abundance (n=3) of bacterial phyla across treatments.** 100A (*A. mangium* in a monospecific plantation system); A(A+E) (mixed plantation of *A. mangium* and *E. grandis*, with sampling at the *Acacia* base; 100E (*E. grandis* in a monospecific plantation system); and E(A+E) (plantation of *A. mangium* and *E. grandis*, with sampling at the *Eucalyptus* base). “Others” represents unclassified sequences.

| **Phylum level** | **100E** | **100A** | **E(A+E)** | **A(A+E)** |
| --- | --- | --- | --- | --- |
| *Proteobacteria* | 26,2 % Ba | 38 % Aa | 39,2 % Aa | 37,1 % Aa |
| *Firmicutes* | 29,1 % Aa | 16,8 % Bb | 13,4 % Bb | 14,8 % Bc |
| *Acidobacteria* | 13,4 % Bb | 12,8 % Bb | 17,1 % Ab | 21,6 % Ab |
| *Bacteroidetes* | 14 % Ab | 14,4 % Ab | 11,8 % Bb | 8,9 % Bd |
| *Actinobacteria* | 5,3 % Ac | 5,8 % Ac | 5,9 % Ac | 6 % Ad |
| *Verrucomicrobia* | 1,5 % Ad | 1,1 % Ad | 1,6 % Ad | 2,2 % Ae |
| *Cyanobacteria* | 1,6 % Ad | 0,6 % Ad | 1,1 % Ad | 0,4 % Ae |
| Other | 3,3 % ^NS^ | 1,9 % ^NS^ | 4,4 % ^NS^ | 4,4 % ^NS^ |

* Averages were compared by Tukey's test (p<0.05). Uppercase letters separate treatments (columns) and lowercase letters, bacterial phyla (lines).
