# Supplementary material for: Shifts in the bacterial community composition along deep soil profiles in monospecific and mixed stands of Eucalyptus grandis and Acacia mangium
Source: PLoS One. 2017 Jul 7;12(7):e0180371. doi: 10.1371/journal.pone.0180371 (PMC5501519; doi:10.1371/journal.pone.0180371)
Supplement: S3 Table — 100A (A. mangium in a monospecific plantation system); A(A+E) (mixed plantation of A. mangium and E. grandis, with sampling at the Acacia base; 100E (E. grandis in a monospecific plantation system); and E(A+E) (plantation of A. mangium and E. grandis, with sampling at the Eucalyptus base). “Others” represents unclassified sequences. (DOCX) [file pone.0180371.s003.docx]

Table S3. **Average** **abundance (n=3) of bacterial phyla in the soil layer.** 100A (*A. mangium* in a monospecific plantation system); A(A+E) (mixed plantation of *A. mangium* and *E. grandis*, with sampling at the *Acacia* base; 100E (*E. grandis* in a monospecific plantation system); and E(A+E) (plantation of *A. mangium* and *E. grandis*, with sampling at the *Eucalyptus* base). “Others” represents unclassified sequences.

| **Phylum level** | **0-100 cm** | **100-300 cm** | **300-500 cm** | **500-700 cm** | **700-800 cm** |
| --- | --- | --- | --- | --- | --- |
| *Proteobacteria* | 20,8 % Cb | 22,4 % Cb | 31,6 % Ba | 46,0 % Aa | 42,3 % Aa |
| *Acidobacteria* | 38,8 % Aa | 31,3 % Aa | 11,7 % Bb | 2,3 % Cd | 2,9 % Cd |
| *Firmicutes* | 6,9 % Cc | 7,0 % C | 31,4 % Aa | 24,9 % Bb | 23,9 % Bb |
| *Bacteroidetes* | 2,1 % ^NS^ | 6,9 % ^NS^ | 14,7 % ^NS^ | 15,4 % ^NS^ | 19,7 % ^NS^ |
| *Actinobacteria* | 4,2 % Bd | 3,7 % Bc | 5,5 % Bc | 8,1 % Ac | 6,9 % Ac |
| *Verrucomicrobia* | 6,3 % Ac | 1,1 % Bc | 0,5 % Bd | 0,3 % Bd | 0,6 % Bd |
| *Chloroflexi* | 1,5 % ^NS^ | 4,4 % ^NS^ | 0,7 % ^NS^ | 0,0 % ^NS^ | 0,9 % ^NS^ |
| *Cyanobacteria* | 0,5 % Ae | 0,1 % Ad | 0,4 % Ad | 2,6 % Ad | 1,0 % Ad |
| *Planctomycetes* | 1,8 % ^NS^ | 0,6 % ^NS^ | 0,4 % ^NS^ | 0,0 % ^NS^ | 0,4 % ^NS^ |
| *Nitrospirae* | 0,1 % ^NS^ | 2,2 % ^NS^ | 0,3 % ^NS^ | 0,0 % ^NS^ | 0,2 % ^NS^ |
| Other | 12,7 % ^NS^ | 4,8 % ^NS^ | 1,2 % ^NS^ | 0,5 % ^NS^ | 0,5 % ^NS^ |

| * Averages were compared by Tukey's test (*p*<0.05). Uppercase letters separate treatments (columns) and lowercase letters, bacterial phyla (lines). |
| --- |
