# Supplementary material for: Shifts in the bacterial community composition along deep soil profiles in monospecific and mixed stands of Eucalyptus grandis and Acacia mangium
Source: PLoS One. 2017 Jul 7;12(7):e0180371. doi: 10.1371/journal.pone.0180371 (PMC5501519; doi:10.1371/journal.pone.0180371)
Supplement: S4 Table — 100A (A. mangium in a monospecific plantation system); A(A+E) (mixed plantation of A. mangium and E. grandis, with sampling at the Acacia base; 100E (E. grandis in a monospecific plantation system); and E(A+E) (plantation of A. mangium and E. grandis, with sampling at the Eucalyptus base). “Others” represents unclassified sequences. (DOCX) [file pone.0180371.s004.docx]

Table S4. **Average** **abundance (n=3) of bacterial classes across treatments.** 100A (*A. mangium* in a monospecific plantation system); A(A+E) (mixed plantation of *A. mangium* and *E. grandis*, with sampling at the *Acacia* base; 100E (*E. grandis* in a monospecific plantation system); and E(A+E) (plantation of *A. mangium* and *E. grandis*, with sampling at the *Eucalyptus* base). “Others” represents unclassified sequences.

| **Classe level** | **100E** | **100A** | **E(A+E)** | **A(A+E)** |
| --- | --- | --- | --- | --- |
| *Betaproteobacteria* | 13,1 % Bb | 24,1 % Aa | 27,0 % Aa | 20,4 % Aa |
| *Bacilli* | 28,5 % Aa | 18,9 % Ba | 11,6 % Cb | 9,6 % Cb |
| *Flavobacteriia* | 8,7 % Bc | 14,3 % Ab | 11,2 % Ab | 8,2 % Bb |
| *Acidobacteriia* | 5,7 % Bc | 5,5 % Bc | 9,3 % Ab | 10,9 % Ab |
| *Alphaproteobacteria* | 4,6 % Bcb | 5,9 % Ac | 7,3 % Abc | 5,8 % Ac |
| *Gammaproteobacteria* | 7,6 % Ac | 3,7 % Bbc | 3,6 % Bc | 3,3 % Bc |
| *Clostridia* | 0,5 % ^NS^ | 1,9 % ^NS^ | 1,7 % ^NS^ | 5,2 % ^NS^ |
| *Deltaproteobacteria* | 1,0 % Ad | 2,3 % Ad | 1,4 % Ad | 2,4 % Ad |
| *Solibacteres* | 1,7 % Ad | 0,9 % Ad | 1,6 % Ad | 2,2 % Ad |
| *Sphingobacteriia* | 4,5 % ^NS^ | 0,0 % ^NS^ | 0,1 % ^NS^ | 0,2 % ^NS^ |
| *Spartobacteria* | 1,2 % Ad | 0,7 % Ad | 1,0 % Ad | 1,4 % Ad |
| Other | 3,3 % Ad | 1,9 % Bd | 4,4 % Ac | 4,4 % Ac |

* Averages were compared by Tukey's test (p<0.05). Uppercase letters separate treatments (columns) and lowercase letters, bacterial phyla (lines).
