# Supplementary material for: Shifts in the bacterial community composition along deep soil profiles in monospecific and mixed stands of Eucalyptus grandis and Acacia mangium
Source: PLoS One. 2017 Jul 7;12(7):e0180371. doi: 10.1371/journal.pone.0180371 (PMC5501519; doi:10.1371/journal.pone.0180371)
Supplement: S5 Table — 100A (A. mangium in a monospecific plantation system); A(A+E) (mixed plantation of A. mangium and E. grandis, with sampling at the Acacia base; 100E (E. grandis in a monospecific plantation system); and E(A+E) (plantation of A. mangium and E. grandis, with sampling at the Eucalyptus base). “Others” represents unclassified sequences. (DOCX) [file pone.0180371.s005.docx]

Table S5. **Average** **abundance (n=3) of bacterial classes in the soil layer.** 100A (*A. mangium* in a monospecific plantation system); A(A+E) (mixed plantation of *A. mangium* and *E. grandis*, with sampling at the *Acacia* base; 100E (*E. grandis* in a monospecific plantation system); and E(A+E) (plantation of *A. mangium* and *E. grandis*, with sampling at the *Eucalyptus* base). “Others” represents unclassified sequences.

| **Classe level** | **0-100 cm** | **100-300 cm** | **300-500 cm** | **500-700 cm** | **700-800 cm** |
| --- | --- | --- | --- | --- | --- |
| *Betaproteobacteria* | 2,9 % Dd | 9,2 % Cb | 21,2 % Ba | 35,8 % Aa | 31,8 % Aa |
| *Bacilli* | 6,7 % Bc | 6,2 % Bc | 24,6 % Aa | 24,6 % Ab | 20,6 % Ab |
| *Acidobacteriia* | 19,0 % Aa | 14,4 % Ba | 7,8 % Cc | 1,1 % Df | 0,1 % Ef |
| *Flavobacteriia* | 1,4 % Cd | 5,7 % Bc | 11,7 % Ab | 14,7 % A | 16,6 % Ac |
| *Alphaproteobacteria* | 11,1 % Ab | 5,0 % Bc | 4,5 % Bd | 4,1 % Bc | 5,6 % Bd |
| *Actinobacteria* | 3,2 % Ccd | 2,9 % Cd | 5,1 % Bd | 8,0 % Ad | 6,3 % Ad |
| *Gammaproteobacteria* | 4,5 % Ac | 4,4 % Ac | 4,9 % Ad | 5,4 % Ae | 3,9 % Ae |
| *Deltaproteobacteria* | 2,3 % Bd | 3,9 % Ac | 1,1 % Be | 0,7 % Bf | 1,0 % Bf |
| *Sphingobacteriia* | 0,3 % ^NS^ | 0,5 % ^NS^ | 2,3 % ^NS^ | 0,6 % ^NS^ | 2,5 % ^NS^ |
| Other | 12,7 % ^NS^ | 4,8 % ^NS^ | 1,2 % ^NS^ | 0,5 % ^NS^ | 0,5 % ^NS^ |

| * Averages were compared by Tukey's test (p<0.05). Uppercase letters separate treatments (columns) and lowercase letters, bacterial phyla (lines). |
| --- |
