# Supplementary figures and images for: Shifts in the bacterial community composition along deep soil profiles in monospecific and mixed stands of Eucalyptus grandis and Acacia mangium
Source: PLoS One. 2017 Jul 7;12(7):e0180371. doi: 10.1371/journal.pone.0180371 (PMC5501519; doi:10.1371/journal.pone.0180371)

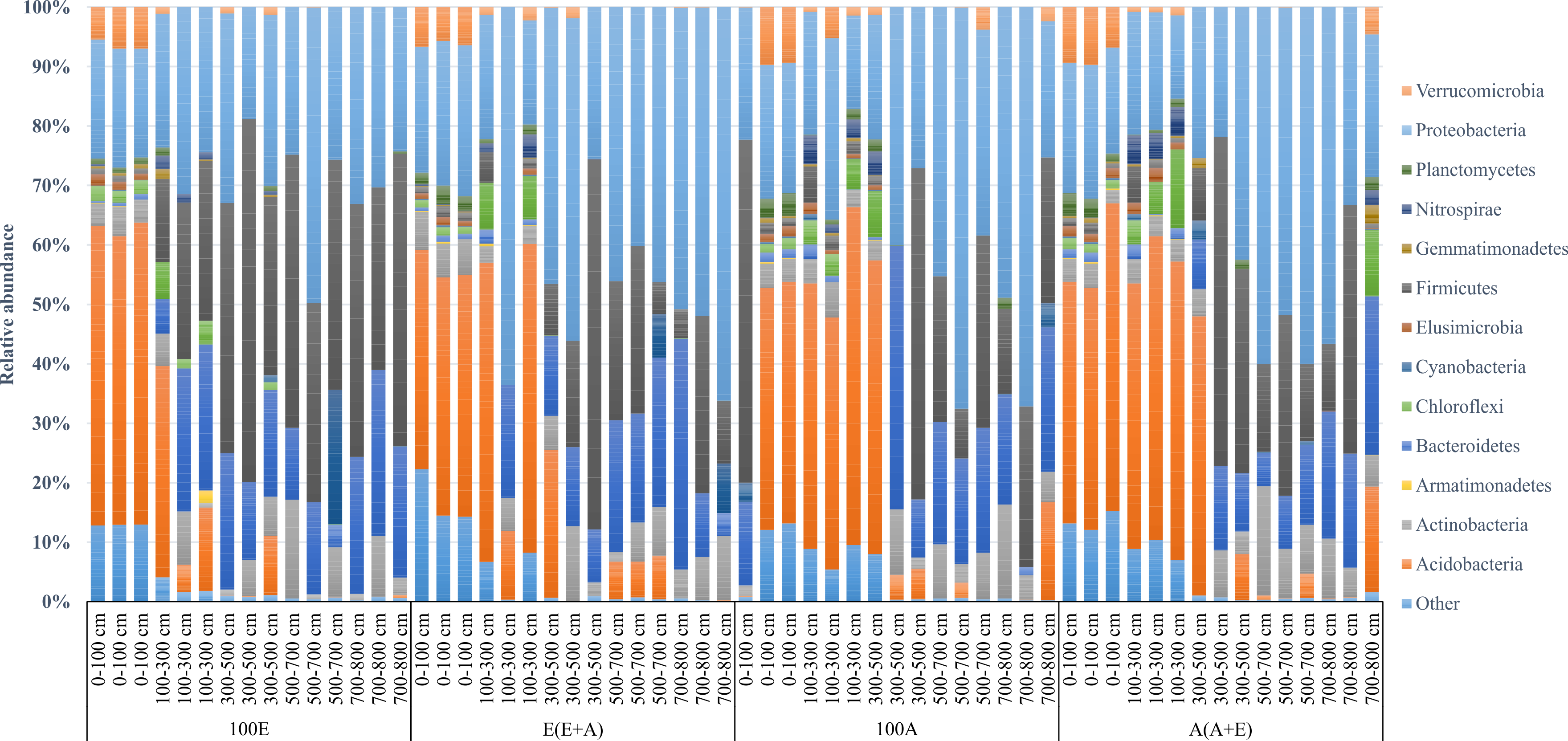

Supplement: S1 Fig — 100A (A. mangium in a monospecific plantation system); A(A+E) (mixed plantation of A. mangium and E. grandis, with sampling at the Acacia base); 100E (E. grandis in a monospecific plantation system); and E(A+E) (plantation of A. mangium and E. grandis, with sampling at the Eucalyptus base). “Others” represents unclassified sequences. Asterisks indicate significant differences (p<0.05) between treatments. (PNG) [file pone.0180371.s006.png]

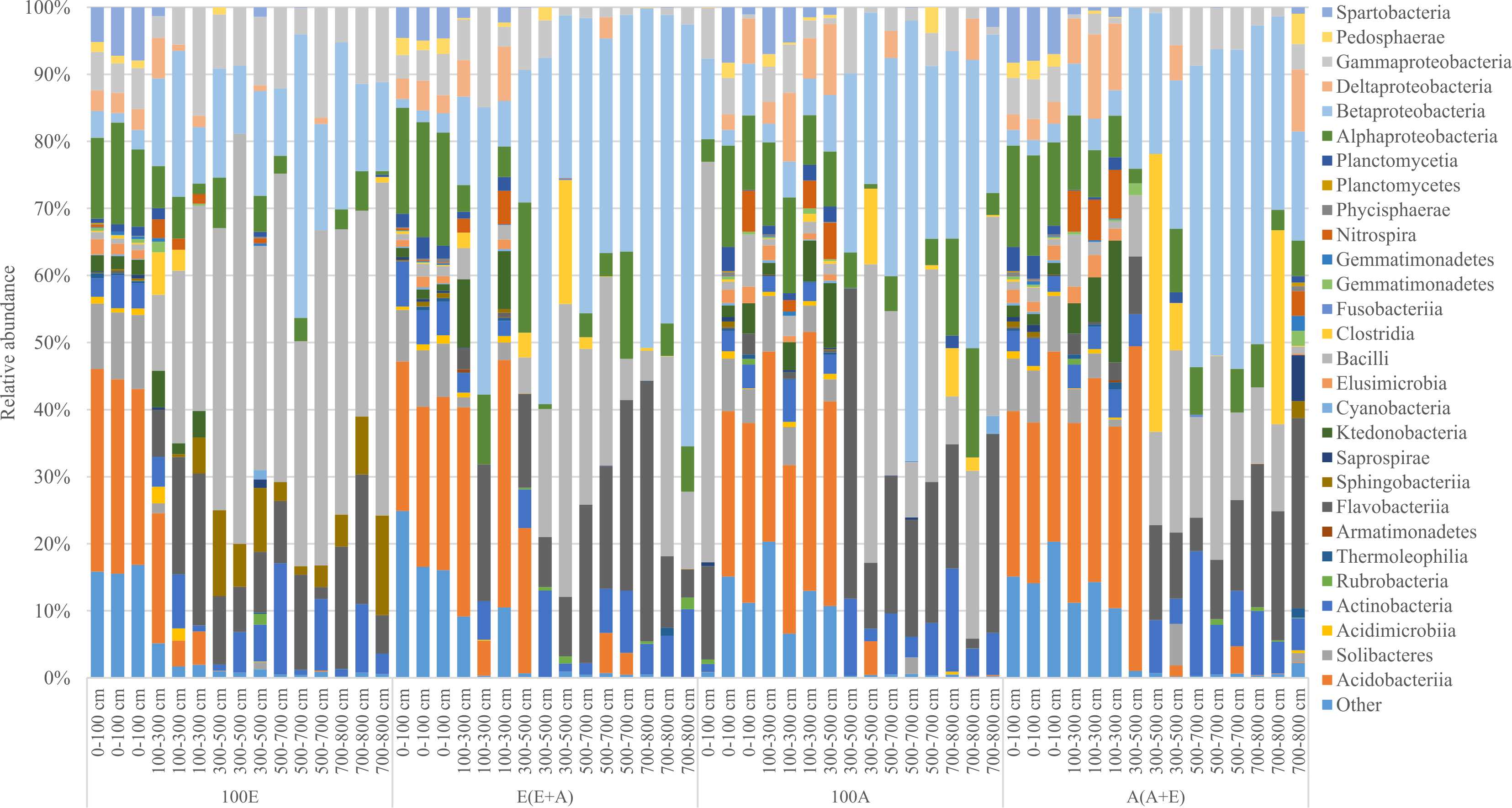

Supplement: S2 Fig — 100A (A. mangium in a monospecific plantation system); A(A+E) (mixed plantation of A. mangium and E. grandis, with sampling at the Acacia base); 100E (E. grandis in a monospecific plantation system); and E(A+E) (plantation of A. mangium and E. grandis, with sampling at the Eucalyptus base). “Others” represents unclassified sequences. (PNG) [file pone.0180371.s007.png]
